# Systematic Review: Impact of Social Determinants of Health on the Management and Prognosis of Gallstone Disease

**DOI:** 10.1089/heq.2022.0063

**Published:** 2022-10-27

**Authors:** Benoît Dupont, Olivier Dejardin, Véronique Bouvier, Marie-Astrid Piquet, Arnaud Alves

**Affiliations:** ^1^ Departement d'Hepato-Gastroenterologie et Nutrition, UNICAEN, CHU de Caen Normandie, Normandie Univ, Caen, France.; ^2^ “Anticipe” U1086 INSERM-UCBN, “Cancers & Preventions,” Team Labelled “League Against Cancer,” UNICAEN, Normandie Univ, CAEN, France.; ^3^ Registre des Tumeurs Digestives du Calvados, “Anticipe” U1086 INSERM-UCBN, UNICAEN, Normandie Univ, Caen, France.; ^4^ Service de Chirurgie Digestive, UNICAEN, CHU de Caen Normandie, Normandie Univ, Caen, France.

**Keywords:** social class, health care disparities, acute pancreatitis, biliary tract diseases, cholecystectomy

## Abstract

**Background:**

Due to its prevalence, gallstone disease is a major public health issue. It affects diverse patient populations across various socioeconomic levels. Socioeconomic and geographic deprivation may impact both morbidity and mortality associated with digestive diseases, such as biliary tract disease.

**Aim:**

The aim of this systematic review was to review the available data on the impact of socioeconomic determinants and geographic factors on gallstone disease and its complications.

**Methods:**

This systematic review was conducted following Preferred Reporting Items for Systematic Reviews and Meta-Analyses guidelines. The MEDLINE and Web of Science databases were searched by two investigators to retrieve studies about the impact of income, insurance status, hospital status, education level, living areas, and deprivation indices on gallstone disease. Thirty-seven studies were selected for this review.

**Results:**

Socially disadvantaged populations appear to be more frequently affected by complicated or severe forms of gallstone disease. The prognosis of biliary tract disease is poor in these populations regardless of patient status, and increased morbidity and mortality were observed for acute cholangitis or subsequent cholecystectomy. Limited or delayed access and low-quality therapeutic interventions could be among the potential causes for this poor prognosis.

**Conclusions:**

This systematic review suggests that socioeconomic determinants impact the management of gallstone disease. Enhanced knowledge of these parameters could contribute to improved public health policies to manage these diseases.

## Introduction

Gallbladder and biliary diseases are a major public health issue, affecting >193 million people worldwide in 2019.^1^ Furthermore, gallstone disease is the leading cause of hospitalization due to gastrointestinal concerns in Western countries.^2,3^

Gallstone disease encompasses many diseases ranging from asymptomatic gallbladder stones to acute biliary pancreatitis (i.e., biliary colic, cholecystitis, obstructive jaundice, or acute biliary cholangitis).^2^ The overall prognosis is quite favorable, with an overall mortality rate <0.5% for gallbladder stones, but mortality rate can reach 20–50% for severe types of acute pancreatitis.^4–7^

The treatment of symptomatic gallstone disease is essentially based on surgical or interventional procedures such as cholecystectomy or endoscopic retrograde cholangiopancreatography (ERCP).^2^ These procedures can be distributed with a certain heterogeneity over the territories and this geographic heterogeneity can condition the access to care. Similarly, medical expertise and offer can differ depending on the care center. Consequently, treatment decisions for the same disease can vary.

Among nonclinical determinants, socioeconomic and geographic deprivation could impact both morbidity and mortality of digestive diseases.^8–12^ Socioeconomic determinants and geographic factors are highly correlated.^13^ However, the geographic factors that influence health are not limited to the material deprivation of the patient's neighborhood, but include determinants such as the distance between patient's home and the health center, the geographical distribution of health centers, and geographical distribution of medical experts.

The aim of this work is to conduct an exhaustive review of the literature that evaluates the impact of nonclinical determinants (socioeconomic or geographic inequalities) on the management and prognosis of gallstone disease and its complications (i.e., cholecystitis, acute cholangitis, acute pancreatitis).

## Methods

### Study selection

Articles included in the review were selected using MEDLINE and Web of Science databases using the following MeSH terms: socioeconomic status (SES), social classes, socioeconomic factors, poverty areas, health care disparities, health care access, pancreatitis, gallstone, cholelithiasis, acute cholangitis, cholecystitis, biliary tract diseases, ERCP, and cholecystectomy and the formula ([Social class OR Poverty areas OR Health care disparities OR Health care access OR SES OR Socioeconomic factors] AND [Pancreatitis OR Gallstone OR Cholelithiasis OR Acute cholangitis OR Cholecystitis OR Biliary tract diseases OR ERCP OR cholecystectomy]) NOT Cancer. Selection was restricted to English-language articles indexed from database inception to October 4th, 2021. We also excluded articles published before 1985.

The search retrieved 102 abstracts that were carefully reviewed by a gastroenterologist (B.D.) and an epidemiologist (O.D.) for clinical relevance. The bibliographies of all full text articles selected were manually searched to identify additional studies that might be relevant. The data extraction process was conducted by a B.D. and verified by an O.D.

### Definition of SES

The definition of SES and its specific assessment varied significantly between articles. Its relevance could be deeply influenced by the country in which the study is conducted. Overall, we can distinguish between two main categories of indicators for SES:

- Some studies used unique and individual variables such as income, socioprofessional category, or insurance status. Studies based in the United States can include and examine race/ethnicity as a social determinant of health. These parameters are not considered in European studies. Studies considering only a race/ethnicity criterion without another variable were excluded.
- Others used collective indicators such as ecological scores that combine different parameters that better assess the complexity of SES. These indicators do not reflect the patients' situations individually, but assimilate their situation to a collective index depending on their residential area. Some studies considered the hospital status and location, urban or rural residential area, or different country regions that can impact access to health care.

### Inclusion criteria

We therefore considered the full text articles that studied the impact of income, insurance status, hospital status, level of education, area of residency, and/or deprivation index in gallstone diseases (gallbladder stones, biliary colic, cholecystitis, obstructive jaundice, acute biliary cholangitis, or acute biliary pancreatitis).

### Exclusion criteria

Studies were excluded if they were unavailable in English. Poster or oral presentation abstracts not linked to full-text articles were also excluded. We excluded studies that exclusively considered race/ethnicity criteria. Indeed, race/ethnicity criteria are not registered in medical studies in the majority of countries, except the United States. This point can lead to difficulties of comparison between studies, especially between Europe and the United States. Second, although there is a strong correlation between race and SES, it has been shown that racial disparities in health status are due to other parameters than only social disparities.^14^ Racial segregation can lead to difference in social/environmental exposures and in care access. Many confounding factors between race and SES exist. We also excluded studies on malignant diseases or acute pancreatitis without data on biliary pancreatitis.

### Outcomes measures

1. Do socioeconomic determinants or geographic factors impact the risk of developing a gallstone disease?
2. Do socioeconomic determinants or geographic factors impact access to treatment for gallstone disease?
3. Do socioeconomic determinants or geographic factors impact quality of care for gallstone disease?
4. Do socioeconomic determinants or geographic factors impact the prognosis of gallstone disease?

### Quality assessment

To elaborate this systematic review, we followed 2020 Preferred Reporting Items for Systematic Reviews and Meta-Analyses (PRISMA) guidelines.^15^ The quality of the studies was evaluated using STROBE criteria.^16^ The items on the STROBE checklist v4 were interpreted in terms of their appropriateness of design to answer the study question. Scores were summarized as 0–11=III, 12–17=II, and 18–22=I, with “I” representing the highest quality studies (Supplementary Table S1). Only the studies with sufficient estimated quality were kept for this review. The review was not registered.

## Results

### Study selection and characteristics

**FIG. 1.**
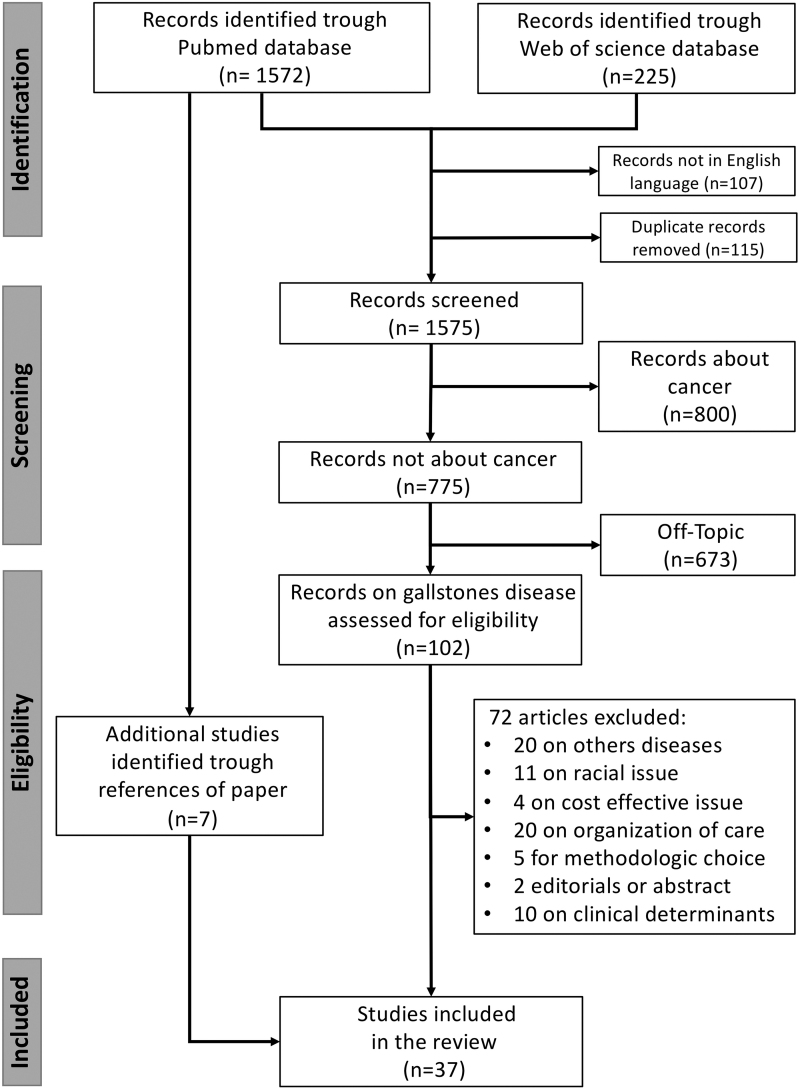
Flowchart describing the study selection process.

Finally, 37 suitable studies were identified for review: 20 based on populations from the United States, 5 from the United Kingdom, 3 from Taiwan, 2 from Italy, and 1 from Switzerland, South Korea, Argentina, China, Sweden, Canada, and the Netherlands (Fig. 1).

Table 1 shows the studies selected by summarizing the study period, its location, the number of included patients, the type of variable used to express the patients' SES, the disease or therapeutic intervention studied, and the main result of the study.

**Table 1.** Studies Describing the Impact of Socioeconomic Status on Gallstone Disease

Study	Country	Period of study	Population (n)	Disease or therapeutic act	Type of variables for SES	Calculation of SES	Results
La Vecchia et al^18^	Italy	1983	2116	Cholelithiasis	Individuals	Education level	The estimated RR of cholelithiasis was substantially below for more educated people (RR=0.65, 95% CI: 0.56–0.75)
Chaturvedi and Ben-Shlomo^17^	United Kingdom	1981–1982	2938	Cholecystectomy	Individuals	Occupation and partner's occupation	Operation ratio was unrelated to deprivation score
Westert et al^26^	Netherlands	1999	24,988	Cholecystectomy	Collective	Community income, degree of urbanization	Men from a low-income community received more cholecystectomies (1.12). Women received less cholecystectomies (0.87)
Roberts et al^25^	United Kingdom	1998–2003	52,096	Pancreatitis	Collective	Index of multiple deprivation, government office regions of England	The incidence of acute pancreatitis was strongly associated with social deprivation 28.4 per 100,000 population in the most deprived quintile vs. 17.2 in the least deprived. Age-adjusted case fatality was also significantly associated with social deprivation (*p*=0.015 at 30 days and *p*=0.017 at 60 days)
Momiyama et al^19^	Taiwan	1999–2001	453; 151 cases, 302 controls	Intrahepatic stone	Individuals	Highest formal education	Increasing level of education lowered the risk of intrahepatic stone (trend *p*=0.004 for men and <0.0001 for women)
Ellis et al^23^	United Kingdom	September 2006—March 2007	963	Pancreatitis	Collective	Index of multiple deprivation	The incidence of acute pancreatitis was highest in the most deprived areas comparing the least deprived with the most deprived area (OR=2.40, 95% CI: 1.94–2.98). The annual age-standardized mortality was 1.2 per 100,000 for least deprived group and 4.4 for most deprived group (*p*=0.044)
Varela and Nguyen^42^	United States	2005–2009	112,540	Cholecystectomy and appendicectomy	Individuals	Insurance status	Univariate analysis showed that commercial/private payer status (OR=1.25, 95% CI: 1.21–1.29) increased the likelihood that a laparoscopic approach would be used for cholecystectomy
Poulose et al^39^	United States	2007	111,021	Choledocholithiasis	Collective	NCHS urban–rural classification scheme for counties	Comparisons across NCHS classes revealed higher proportions of ERCP in urban areas (NCHS 1–4), while a higher proportion of common bile duct exploration was seen in rural areas (NCHS 5–6). ERCP availability was high in metropolitan areas (available in 35–44% of hospitals NCHS 1–4) and low in rural areas (25% of NCHS 5 hospitals and 5% NCHS 6). Percutaneous management was similar across NCHS classes
Greenstein et al^27^	United States	1998–2008	843,179; 200,000 matched patients	Cholecystitis	Individuals	Insurance status	While 89% of the private insurance cohort underwent cholecystectomy during their hospitalization, only 83% of the Medicaid population received equivalent care (*p*=0.001). The Medicaid cohort also had reduced rates of laparoscopic surgery (78% vs. 69%, *p*=0.001) and an increased conversion rate from laparoscopic to open surgery (3.9% vs. 3.0%, *p*=0.001)
Neureuther et al^48^	United States	2006–2009	1090	Cholecystectomy	Individuals	Insurance status	Uninsured patients were much more likely to have emergency surgery (99.3% vs. 47.9%, *p*<0.001, χ^2^)
Petrelli et al^28^	Italy	2006–2008	14,083	Cholecystectomy	Individuals	Education level	Subjects with a lower education level registered for cholecystectomy have access to surgery at a rate 16.2% lower than for subjects with a higher education. The estimated difference between lower and higher education in terms of median waiting time decreases to 13.5% for cholecystectomy
McNabb-Baltar et al^53^	United States	1998–2009	248,942	Cholangitis	Individuals	Insurance status	Medicaid and Medicare patients were more likely to die (OR=1.64, *p*=0.001; OR=1.24, *p*=0.001), to experience a prolonged LOS (OR=1.74, *p*=0.001; OR=1.25, *p*=0.001), and to incur high HC (OR=1.23, *p*=0.002; OR=1.12, *p*=0.002) compared to privately insured patients
Chang et al^21^	Korea	1981–2010	7949	Cholecystectomy	Collective	Engel's coefficient	Engel's coefficient was strongly correlated with changes in the proportion of the common bile duct stone group (*r*=0.980, *p*<0.001)
Roberts et al^24^	United Kingdom	1999–2010	10,589	Pancreatitis	Collective	Index of multiple deprivation; welsh index of multiple deprivation	The incidence of acute pancreatitis was 1.9 times higher (95% CI: 1.8–2.0) among the most deprived quintile of patients compared with the most affluent quintile
Hanmer et al^38^	United States	2010	55,863	Biliary tract disease	Individuals	Insurance status	Uninsured patients were statistically significantly less likely to be transferred when compared to privately insured patients (OR=0.73, 95% CI: 0.55–0.96, *p*=0.024)
Roberts et al^55^	United Kingdom	1999–2010	10,589	Pancreatitis	Collective	Index of multiple deprivation; welsh index of multiple deprivation	No significant associations overall between social deprivation and mortality for acute pancreatitis
Loehrer et al^29^	United States	2001–2009	141,344	Cholecystitis	Individuals	Insurance status	Before the 2006 reform, government-subsidized/self-pay patients had a 6.6–9.9 percentage-point lower (*p*<0.001) probability of immediate cholecystectomy in both MA control states. The MA insurance expansion was independently associated with a 2.5 percentage-point increased probability of immediate cholecystectomy for all GS/SP patients in MA (*p*=0.049)
Palsson and Sandblom^41^	Sweden	2005–2009	39,333	Cholecystectomy	Individuals	Income, education level, marital status, Country of Birth	Neither race/ethnicity background, marital status, level of education, or income level had any significant impact on the incidence of surgery
Compagnucci et al^22^	Argentina	2014	114; 49 cases–65 controls	Gallstone disease	Individuals	Personal structured questionnaire	No difference was found between cases and controls according to SES
Mador et al^36^	Canada	2001–2011	4287	Early cholecystectomy after sphincterotomy	Collective	Neighborhood income	The proportion of patients undergoing early cholecystectomy was significantly different between groups based on socioeconomic quintile (*p*=0.0134). Rates ranged from 41.3% (highest quintile) to 48.4% (second lowest quintile)
Ibrahim et al^50^	United States	2009–2013	583,991	Cholecystectomy	Collective	Hospital status (“critical or non critical access hospitals”)	Critical access hospitals had lower rates of in-hospital mortality (1.3% vs. 2.2%, aOR=0.58, 95% CI: 0.47–0.72, *p*<0.001), lower rates of serious complications (5.0% vs. 12.1%, OR=0.32, 95% CI: 0.28–0.36, *p*<0.001) and overall complications (13.2% vs. 21.7%, OR=0.48, 95% CI: 0.45–0.52, *p*<0.001)
Ambur et al^43^	United States	2005–2011	2,058,611	Cholecystectomy	Individuals	Household income, insurance status	Patients with higher income had lower mortality risk (OR=0.88, 95% CI: 0.82–0.95, *p*<0.001)
Lu et al^52^	Taiwan	2003–2012	11,184	Percutaneous cholecystostomy	Individuals	Criteria of Taiwan's NHI database	After percutaneous cholecystostomy, in-hospital mortality was significantly higher in the low-income population group than in the global population (OR=1.816, 95% CI: 1.079–3.056)
Kang et al^20^	China	June 2012–August 2012	21,435	Gallbladder diseases	Individuals	Area of residence, education level, family income	Multivariate logistic regression analysis showed that people living in rural areas (OR=1.65, 95% CIs: 1.49–1.82) were associated with gallbladder diseases
Lu et al^51^	Taiwan	2003–2012	225,558	Cholecystectomy	Individuals	Criteria of Taiwan's NHI database	Patients from low-income population showed higher rates of 30-day mortality (LIP: 4.65% vs. GP: 2.18%, *p*<0.001), in-hospital complications (LIP: 5.62% vs. GP: 4.01%, *p*=0.008), and readmission for complications (LIP: 1.83% vs. GP: 1.09%, *p*<0.001)
Bhutiani et al^31^	United States	2011–2016	103,838	Cholecystectomy	Individuals	Insurance status	After Medicaid expansion, patients were more likely to have their operation performed as an outpatient (80.0% vs. 78.2%, *p*<0.001). They were more likely to have Medicaid (33.9% POST vs. 15.0% PRE) and were less likely to be uninsured (0.3% POST vs. 2.7% PRE) or self-payers (2.0% POST vs. 8.7% PRE; *p*<0.001)
Carmichael et al^45^	United States	January 2018–June 2018	289	Cholecystectomy	Collective	SVI	On multivariable analysis, adjusting for chronicity of symptoms and patient proximity to the hospital, having high SVI (>70th percentile) was associated with higher odds of undergoing an emergent vs. an elective procedure (OR=2.05, *p*=1/4 0.04)
Huang et al^54^	United States	2009–2014	68,642	Post-ERCP unplanned hospital encounters	Collective/individuals	Insurance status/volume hospital	Lack of insurance is associated with unplanned hospital encounters (OR=1.18, CI: 1.06–1.32) in multivariable analysis. In the fully adjusted model, facilities performing >300 ERCPs per annum demonstrated a 25% reduction in odds of an adverse event compared to facilities performing <50 ERCPs per annum
Moore et al^47^	United States	January 2018–June 2018	287	Cholecystectomy	Individuals	Residential address, marital status, primary language and interpreter needs, documentation of a PCP, and insurance status	After multivariable regression, lack of a PCP was a significant predictor of emergent presentation (OR=5.02, *p*<0.001) as was public or no insurance compared with private insurance (OR=2.78, *p*<0.001)
Carmichael et al^46^	United States	January 2018–September 2018	366	Cholecystectomy	Collective	SVI, ADI, CNI, DCI	On multivariable modeling, patients with high social vulnerability were more likely to undergo emergency surgery compared with those with low social vulnerability in accordance with all four scales: SVI (OR=3.24, *p*<0.001), ADI (OR=3.2, *p*<0.001), CNI (OR=1.90, *p*=1/4 0.04), and DCI (OR=2.01, *p*=1/4 0.03). The scales all had comparable predictive value
Schneider et al^44^	Switzerland	1997–2018	57,788	Cholecystectomy	Individuals	Area of residence, insurance status	Multivariate analyses identified lack of private insurance (*p*≤0.01) as well as rural residence (*p*≤0.01) with impaired access to minimally invasive surgery
Godat et al^30^	United States	2012–2015	189,023	Cholecystectomy	Collective/individuals	Insurance status, hospital status, ZIP code, income quartile	There was a decrease in the rate of emergency cholecystectomies after implementation of the ACA; pre-ACA 62.1% to post-ACA 59.3%, *p*<0.001). Admissions to teaching hospitals increased in the post-ACA period, increasing from 45.4% to 60.4% of all admissions for acute gallbladder disease (*p*<0.001). In the post-ACA period, the payer distribution for admissions decreased for self-pay (19.3–13.6%, *p*<0.001), Medicaid increased (26.3–34.0%, *p*<0.001)
McCarty et al^37^	United States	2008–2014	1,492,877	Percutaneous cholecystostomy vs. cholecystectomy	Individuals	Insurance status, income	Multivariable regression demonstrated multiple socioeconomic factors as Medicare payer status and household income influencing the utilization of percutaneous cholecystectomy (all *p*<0.001)
Janeway et al^49^	United States	2011–2014	321,335	Cholecystectomy	Individuals	Area of residence, insurance status, income	The odds of undergoing ambulatory vs. inpatient cholecystectomy were significantly lower in Medicare (aOR=0.77, 95% CI: 0.75–0.80, *p*<0.001), Medicaid (aOR=0.56, 95% CI: 0.54–0.57, *p*<0.001), and uninsured/self-pay (aOR=0.28, 95% CI: 0.27–0.28, *p*<0.001) patients relative to privately insured patients. Patients with Medicaid and those classified as self-pay/uninsured had higher odds of post-operative complications and unplanned admission. Residence in large metropolitan areas was associated with significantly lower odds of intraoperative and post-operative complications (aOR=0.44, 95% CI: 0.31–0.62, *p*<0.001, and aOR=0.79, 95% CI: 0.65–0.96, *p*=0.016, respectively)
Chouairi et al^35^	United States	2008–2014	205,012	Gallstone pancreatitis	Individuals	Insurance status, income	Multivariable regression demonstrated Medicare payer status and household income decreased the odds of undergoing same admission cholecystectomy and ERCP (all *p*<0.001)
Kabaria et al^40^	United States	2005–2014	105,433	ERCP in acute biliary pancreatitis	Collective/individuals	Hospital status and volume, area of residence, insurance status	High ERCP volume hospitals, teaching hospitals, and hospitals in the Midwest and West were more likely to perform urgent ERCP
Shmelev et al^34^	United States	2000–2014	578,258	Same admission cholecystectomy for mild acute pancreatitis	Individuals	Hospital status and location, insurance status	In multivariate analysis, same-admission cholecystectomy was positively associated with private insurance (vs. Medicare; OR=1.1, 95% CI: 1.0–1.3), and large (vs. small; OR=1.3, 95% CI: 1.2–1.4) urban hospitals (vs. rural; OR=1.5, 95% CI: 1.3–1.7) of the South (vs. Northeast; OR=1.5, 95% CI: 1.3–1.7)

ACA, Affordable Care Act; ADI, area deprivation index; aOR, adjusted odds ratio; CI, confidence interval; CNI, community needs index; DCI, distressed communities index; ERCP, endoscopic retrograde cholangiopancreatography; GP, general population; GS/SP, Government-Subsidized/Self-Pay; HC, hospital charges; LIP, low-income population; LOS, length of stay; MA, Massachusetts; NCHS, National Center for Health Statistics; NHI, National Health Insurance; OR, odds ratio; PCP, primary care provider; RR, relative risk; SES, socioeconomic status; SVI, Social Vulnerability Index.

Given the heterogeneity of indices used to assess the patients' SES and the diversity of outcomes described in these studies, we were unable to perform a meta-analysis on this issue.

### Do social determinants impact the risk of developing a gallstone disease?

Old data seemed to show a higher prevalence of gallstone disease in the most socially deprived patients. A study conducted in England in the 1990s showed a standardized consultation rate for gallstone disease >100%, which is higher than the expected proportion, in the most disadvantaged strata of the population; this finding suggests a possible overincidence, but without significant difference among the rest of the population.^17^ One study shows an estimated relative risk (RR) of cholelithiasis substantially lower among highly educated people in comparison to patients with only primary school education (RR=0.65, 95% confidence interval [CI]: 0.56–0.75).^18^

A case–control study aimed at identifying the risk factors associated with intrahepatic stones showed that a higher level of education lowered the risk of intrahepatic stones.^19^ More recently, a study conducted in Jilin Province showed that patients with gallbladder diseases were more often from a rural area and had a lower level of education and lower income.^20^ In a multivariate analysis, only living in a rural area was significantly associated with gallbladder diseases (odds ratio [OR]=1.65, 95% CI: 1.49–1.82).^20^

A study conducted in South Korea over 30 years showed an increase in the proportion of gallbladder stones, following in the same proportions, the improvement in socioeconomic conditions in the country.^21^ It is difficult to determine whether the increased prevalence of gallstone disease was due to an increased incidence of lithiasis or to an increased diagnosis correlated with an improvement in the performance and accessibility of paraclinical examinations such as ultrasound. Likewise, this study could not determine whether, at the individual level, social deprivation influenced the risk of gallstone disease. A case–control study indirectly questions the disproportionate risk of gallstone disease as related to SES. This study did not find any difference depending on the SES.^22^ This work was not designed to truly answer the question of the incidence of gallstone disease according to the populations' socioeconomic determinants.

In the specific case of biliary pancreatitis, more robust data exist. Socioeconomic deprivation has been associated with increased incidence of acute pancreatitis.^23,24^ In a large British study of 10,589 cases of acute pancreatitis, Roberts et al^24^ reported an incidence 1.9 times (95% CI: 1.8–2.0) higher in severely disadvantaged patients than in the highly affluent patients. This difference persisted when we considered only gallstone etiology (1.5, 95% CI: 1.4–1.7), even if it was more obvious for alcoholic acute pancreatitis (3.9, 95% CI: 3.4–4.5).^24^ These data confirmed the results of a preliminary study from the same team^25^ and of another earlier British study of 963 cases, which also found a higher incidence of biliary pancreatitis in severely disadvantaged people.^23^

Overall, the data currently seem too limited to conclude the potential impact of socioeconomic determinants on the incidence of gallstone disease, except for biliary pancreatitis.

### Do social determinants impact access to treatment for gallstone disease?

All the recent studies conclude that there is rather limited access to cholecystectomy for the weakest social categories. A Dutch study shows an impact of income level on the incidence of cholecystectomy without analyzing the cause of these differences. They report a decreased incidence in low-income women (0.87) and an increase in men (1.12).^26^ After acute cholecystitis, it has been demonstrated that more patients with private insurance underwent cholecystectomy during the same hospitalization than patients with Medicaid (89% vs. 83%; *p*<0.001).^27^ The time to access cholecystectomy is inversely associated with the level of education: patients with a low level of education had a 16.2% lower rate of access to surgery and a 13.5% longer waiting time.^28^

This limited access to cholecystectomy for patients with a low SES is also illustrated through changes in cholecystectomy rates before and after health care reform in the United States. Before reform in Massachusetts in 2006, patients with government insurance and no insurance had 6.6% and 9.9% lower chances of having an immediate cholecystectomy after an episode of acute cholecystitis, respectively. The health care reform resulted in a 2.5% independent improvement of this probability in these same patients (*p*=0.049).^29^

Conversely, the National Reform of 2014 allowed a reduction in emergency cholecystectomies (62.1–59.3% after the reform, *p*<0.01), an increase in the proportion of patients treated in teaching hospitals (45.4–60.4%; *p*<0.01), and an increase in patients receiving Medicaid (26.3–34.0%, *p*<0.001) inversely proportional to the decrease in self-pay patients (19.3–13.6%, *p*<0.001).^30^ This decrease in the proportion of self-pay patients was also observed in a cohort study on cholecystectomies performed for benign gallbladder disease.^31^ In addition, there was an increase in the proportion of outpatient surgeries (80.0% vs. 78.2%, *p*<0.001).^31^

While it is clearly established that there is a benefit to perform cholecystectomy during the same period of hospitalization for nonsevere acute biliary pancreatitis,^32^ the applicability of this recommendation is sometimes difficult.^33^ A recent large-scale American study showed that the rate of same admission cholecystectomy for nonsevere acute biliary pancreatitis was higher in patients with private insurance (OR=1.1, 95% CI: 1.0–1.3).^34^ Another U.S. study supports these results in concluding that Medicare payer status decreased the odds of undergoing same admission cholecystectomy after ERCP for acute gallstone pancreatitis.^35^

Overall, after a complication (biliary colic, cholecystitis, or biliary pancreatitis), the likelihood of having cholecystectomy, as recommended, is lower in severely deprived patients. However, in patients previously hospitalized for gallstones treated with ERCP, the rate of early cholecystectomy (performed within 14 days after sphincterotomy) was significantly lower in populations of patients with high SES (41.3% vs. 48.4%, *p*=0.0134).^36^ While these data should be interpreted with caution, they appear to show that once treated, patients from a severely deprived class follow the proposed treatments. The main explanation would be that the initial access to treatment is difficult. It is also possible that caregivers take advantage of hospitalization to complete the entire care program for the most fragile or the most isolated patients who would be less able to return for the rest of their care.

These inequities are also observed for access to other therapeutic procedures. Percutaneous cholecystostomy represents an alternative to surgery in clinically frail patients. Its access and use might be impacted by socioeconomic factors.^37^ It has been shown that patients from poor social classes are less easily transferred for biliopancreatic diseases (OR=0.73, 95% CI: 0.55–0.96, *p*=0.024), which would imply inadequate access to specific care techniques or expert centers for these populations.^38^

Inequities could also be due to geographic factors. Regarding the management of bile duct stones, Poulose et al^39^ demonstrated that patients treated in urban areas benefitted more from ERCP, while patients in rural areas were more easily managed by surgery. The availability of ERCP in urban areas was estimated at 35–44% versus 5–25% in rural areas.^39^

Recent data showed that the rate of urgent ERCP performed in the 2000s in patients with acute biliary pancreatitis without associated cholangitis was higher in high-volume hospitals, teaching hospitals, and midwestern and western U.S. states.^40^ Even if the indication for this procedure has changed and is currently reserved for pancreatitis associated with cholangitis, this testifies to the unequal access to urgent ERCP according to institutions. Concerning same admission cholecystectomy for mild acute biliary pancreatitis, a previously cited study shows that same admission cholecystectomy was positively associated with urban hospitals (vs. rural; OR=1.5, 95% CI: 1.3–1.7) of the South (vs. Northeast; OR=1.5, 95% CI: 1.3–1.7).^34^

Only old data did not seem to show an impact of SES on performing cholecystectomy: there was no increase in the standardized rate of surgery according to the SES estimated by the patient's or spouse's profession.^17^ These conclusions were confirmed more recently by the Swedish Palsson.^41^ In this investigation, neither marital status, level of education, or level of income showed any impact on the incidence of surgery. However, it should be noted that this study was conducted on a population of cholecystectomized patients. There is no detail in these data to determine the impact of SES on access to surgery.

All these data, even if they remain open to criticism, highlight more complex access to various therapeutic interventions for vulnerable populations to treat gallstone disease without the potential to identify the factors that influence these conclusions: limited access to care for economic reasons, difference in treatment by physicians, patient refusal of proposed care depending on the level of education, and so on.

### Do social determinants impact quality of care for gallstone disease?

Socioeconomic determinants could also influence the quality of care. Varela and Nguyen^42^ showed that patients with private insurance were more likely to have a laparoscopy than open surgery for cholecystectomy (OR=1.25, 95% CI: 1.21–1.29). These data were confirmed by two American studies. The first shows an increased risk of conversion to open surgery for patients receiving Medicaid (3.9% vs. 3.0%, *p*=0.001).^27^ In the second one, patients with the lowest income level underwent urgent operations more frequently (71.7% vs. 66.9%, *p*<0.001) by the open approach (14.8% vs. 11.3%, *p*<0.001).^43^

A Swiss study concluded that there was an increased risk of open surgery in patients without private insurance.^44^ Similarly, it has been shown that socially frail patients are more likely to undergo cholecystectomy in an emergency setting than electively, regardless of the clinical situation and the cause of the intervention. In two studies, using collective indices of social deprivation, in particular the “Social Vulnerability Index,” Carmichael et al^45,46^ showed that having a high index of deprivation increases the risk of emergency operation (OR=2.05, *p*<0.04). The same team showed that this increased risk was present in patients without insurance or with public insurance (OR=2.78, *p*<0.001).^47^

In addition, they found in this study that these patients had more chronic symptoms, which would tend to show that they wait for long periods of time to consult with a physician and ultimately need emergency surgery.^47^ Another U.S. study showed that uninsured patients were much more likely to undergo urgent operations (99.3% vs. 47.9%, *p*<0.001).^48^ A recent American study showed that the probability of outpatient surgery was lower in patients with Medicare (OR=0.77, 95% CI: 0.75–0.80, *p*<0.001) or Medicaid (OR=0.56, 95% CI: 0.54–0.57, *p*<0.001), or among self-pay patients (OR=0.28, 95% CI: 0.27–0.28, *p*<0.001), than in patients with private insurance.^49^

The type of institution would possibly have little impact on the quality of interventions. According to Ibrahim et al,^50^ “Critical Access Hospitals” in the United States, defined as hospitals with fewer than 25 beds and located more than 35 miles from any other hospital, had a lower in-hospital mortality after cholecystectomy (1.3% vs. 2.2%, OR=0.58, 95% CI: 0.47–0.72, *p*<0.001), lower rates of major complications (5.0% vs. 12.1%, OR=0.32, 95% CI: 0.28–0.36, *p*<0.001), or overall complications (13.2% vs. 21.7%, OR=0.48, 95% CI: 0.45–0.52, *p*<0.001). However, these results must be considered with caution because the patients treated in these centers were less severe.^50^

Conversely, in biliary pancreatitis, it has been shown that hospital status determines the application of best practice recommendations. Thus, the proportion of cholecystectomized patients during the same period of hospitalization for nonsevere biliary pancreatitis was higher when these patients were treated in hospitals in urban areas (OR=1.5, 95% CI: 1.3–1.7) or in high-volume hospitals (OR=1.3, 95% CI: 1.2–1.4).^34^ Finally, the Swiss study, previously cited, demonstrated that patients living in a rural area were more likely to have open surgery for cholecystectomy.^44^

### Do social determinants impact the prognosis of gallstone disease?

Overall, all available data show a poor prognosis for gallstone disease in severely deprived patients regardless of their clinical status.

Patients with low socioprofessional status have poor prognosis after cholecystectomy.^43^ In a large American study of 2,058,611 cholecystectomies, the authors separated patients into 4 categories according to the quartile distribution of household income. The poorest group of patients was younger (50.5 vs. 53.4 years for the richest *p*<0.001), had fewer comorbidities according to the Charlson comorbidity index (2.08 vs. 2.16, *p*<0.001), and had more patients without private medical insurance (31.1% vs. 54.8%, *p*<0.001).^43^ High-income patients had lower mortality (OR=0.88, 95% CI: 0.82–0.95, *p*<0.001), while patients without private insurance had more post-operative complications and poorer survival.^43^

An Asian study on 225,558 cholecystectomies confirmed these results. A higher rate of 30-day mortality (4.65% vs. 2.18%, *p*<0.001), complications (0.62% vs. 4.01%, *p*=0.008), or readmissions for complications (1.83% vs. 1.09%, *p*<0.001) was observed in patients with low income than in the general population.^51^ In outpatients, the rate of complications or rehospitalization was higher in self-pay patients or patients with Medicaid/Medicare.^49^

This poor prognosis is also true in patients treated with percutaneous cholecystostomy, with significantly higher hospital mortality in low-income patients (OR=1.816, 95% CI: 1.079–3.056).^52^

In the United States, patients hospitalized for acute cholangitis with Medicare/Medicaid had a poorer prognosis than insured patients: a significant increase in mortality, a longer hospital stay and increased medical costs.^53^ More generally, in patients who benefitted from ERCP, the patient's lack of insurance was a risk factor for readmittance following this procedure (OR=1.18, CI: 1.06–1.32).^54^

Regarding acute biliary pancreatitis, Roberts et al^55^ did not find a significant impact of socioeconomic deprivation on the mortality of pancreatitis, regardless of its etiology. To our knowledge, no data exist on the influence of these parameters on the occurrence of severe or complicated forms of acute biliary pancreatitis.

Data about the impact of geographical factors on prognosis are few. In one study, the rate of complications or rehospitalization in outpatients after cholecystectomy was lower in patients living in metropolitan areas than in rural areas (OR=0.44, 95% CI: 0.31–0.62, *p*<0.001, and adjusted OR=0.79, 95% CI: 0.65–0.96, *p*=0.016, respectively).^49^

## Discussion

Both severe and complicated types of gallstone disease seem more frequent in underprivileged populations.^23,25,43^ Furthermore, their prognosis seems less favorable regardless of their clinical status.^23,43,51,52^

If this review seems to show an impact of socioeconomic determinants or geographic factors on access to treatment and the prognosis of biliopancreatic disease, the precise role and the importance of these determinants are very difficult to dissect.

Several risk factors influence the occurrence of gallstone disease (sedentary lifestyle, diabetes, obesity, Non-Alcoholic SteatoHepatitis, diet, hormonal treatments, and history of bariatric surgery).^56^ These risk factors are socially stratified and distinguishing the impact of a socioeconomic factor on the disease or its risk factors is not always easy. Likewise, a socially stratified risk factor can influence the development of a severe type of the disease and the type of treatment provided. For example, it is clearly established that the risk of gallbladder stones is higher in patients with obesity, but these patients also have an increased risk of presenting symptomatically or severe type of the disease.^57^ Obesity can also affect the type of surgery (open, outpatient surgery, etc.) and the complication rate.^58^ Obesity is clearly impacted by socioeconomic determinants.^59,60^

The reasons for the association between SES or geographic distribution and lithiasis disease or the quality of its management are currently hypothetical. Further analyses would be necessary to determine the relative impact of each of these determinants on the following: disease development, initial diagnosis, attitudes of patients and caregivers toward the disease, quality of patient care offered and provided, monitoring, and prevention. For example, the lesser quality interventions may be due to a less favorable clinical situation for patients who wait longer to seek medical care.^47^ In addition, caregiver's might tend to choose treatments that reduce care costs or the length of stay for financially fragile patients. Finally, the level of expertise of caregivers in centers caring for these deprived patients might be lower.^54^

The data collected in this systematic review have several limitations. A majority of the studies are conducted in the United States. In this country, where payment for health care costs can depend directly on the patient's insurance status, the level and type of treatment can clearly depend on the patient's SES. This is perfectly illustrated by the changes in cholecystectomy management of U.S. patients before and after the health insurance reform in the United States.^29–31,61^ In European countries, the health care system generally allows for the equal treatment of patients after the diagnosis. Potential inequality according to social status can result from differences in patient access to diagnosis and to the center of competence.^62^ Few data from European countries are currently available to verify the potential transposition and universality of results shown in this review.

On the other hand, the tools used in this review to assess patients' SES are heterogeneous, which can lead to difficult comparisons. The majority of the studies selected in this review uses individual indices such as education level, occupation, income, or insurance status. While these indices are specific to patients, few studies combine a synergistic analysis of several of these indices that could cause interpretation bias. For example, a patient may have low income over a period of time, despite a high level of education. Conversely, other studies use indices of social deprivation, which have the large advantage of providing a measure of people's SES in the absence of individual data by using the patients' address.

However, the place of residence is not necessarily a relevant index to define the patients' economic status. In the future, the use of standardized assessment tools could limit these biases and allow a better comparison of results between studies.^63,64^

A better knowledge of factors influencing the management of biliary diseases could help to restore health equity in the management of gallstone disease. Since patients' insurance status can impact their access to high-quality care, health insurance policies can be changed first as it was conducted with the reform of health insurance in the United States in 2013. In theory, improving care for populations with low SES should be based on improved access to teaching hospitals and/or high-volume care centers.^65–67^ Currently, the geographic distribution of the centers is only slightly regulated.

An example of territorial organization of care has been used for several years to manage acute illnesses by creating neurovascular units to manage stroke or to allow for the early treatment of patients with heart attack, with the distribution of coronary angiography centers. We could imagine, using the same example, a regulated distribution of ERCP centers as a result of the impact of ERCP delays on the prognosis of acute cholangitis.^68,69^ At the patient level, the fight against risk factors (sedentary lifestyle, and obesity), which are themselves socially stratified, can make it possible to reduce the occurrence of gallstones disease, especially in the most fragile patients.

To cite only this example, the creation of walking groups within disadvantaged communities has proven to improve the practice of physical activity.^70^ The support of patients by workers dedicated to this task can improve the care and adherence to care of the most fragile patients. Interprofessional teams that include social workers in integrated care settings can improve the coordination of care and behavioral health of patients, compared to the usual primary care model. The addition of social workers to primary care teams reduced Emergency Department Visits^71^ or the number of hospitalizations.^72^

Following the same logic, patient navigation programs using community-based culturally and linguistically concordant patient navigators, which can serve as a bridge between the patient and the health care system, have been developed for many years to support the most fragile patients and motivate them to follow the best care. Other programs have shown to improve enrollment in cardiac rehabilitation,^73^ colorectal cancer screening participation,^74^ or early access to supportive care for patients with advanced cancer.^75^ Such programs could be developed in gallstone diseases to improve access to care and avoid emergency treatment or complications.

At the level of physicians, interventions aimed at improving adherence to guidelines could be elaborated.^76^ Such interventions could make it possible to develop good surgical practices and standardize these practices regardless of geographical areas or local expertise. Financial incentive strategies have also been developed, but with mixed results.^77^

## Conclusion

Gallstone disease represents a growing public health problem in Western countries. Few data are currently known on the impact of socioeconomic determinants and geographic factors on the occurrence and management of these diseases, but all seem to highlight poor access to optimal treatment for vulnerable populations. A better knowledge of these parameters could possibly improve public health policies for the management of these diseases and the distribution of care.

## Supplementary Material

Supplemental data

Supplemental data

## Abbreviations Used

ACA: Affordable Care Act
ADI: area deprivation index
aOR: adjusted odds ratio
CI: confidence interval
CNI: community needs index
DCI: distressed communities index
ERCP: endoscopic retrograde cholangiopancreatography
GP: general population
LIP: low-income population
MA: Massachusetts
NCHS: National Center for Health Statistics
OR: odds ratio
PCP: primary care provider
RR: relative risk
SES: socioeconomic status
SVI: Social Vulnerability Index
