# Supplementary material for: Systematic Review: Impact of Social Determinants of Health on the Management and Prognosis of Gallstone Disease
Source: Health Equity. 2022 Oct 27;6(1):819–35. doi: 10.1089/heq.2022.0063 (PMC9629913; doi:10.1089/heq.2022.0063)
Supplement: Supplemental data [file Suppl_TableS1.docx]

| **Study** | **Title** | **Introduction** | | **Methods** | | | | | | | | | **Results** | | | | | **Discussion** | | | | | **Summary Quality** | **Primary research question** |
| --- | --- | --- | --- | --- | --- | --- | --- | --- | --- | --- | --- | --- | --- | --- | --- | --- | --- | --- | --- | --- | --- | --- | --- | --- |
|  |  | Background | Objective | Study design | Setting | Participants | Variables | Data sources | Bias | Study size | Quantitative variables | Statistical methods | Participants | Descriptive data | Outcome data | Main results | Other analyses | Key results | Limitations | Interpretation | generalizability | funding |  |  |
| La Vecchia et al.,^18^ | 1 | 1/2 | | 6/9 | | | | | | | | | 3/5 | | | | | 4/5 | | | | | **II** | **Y** |
| Chaturvedi et al.,^17^ | 1 | 2/2 | | 5/9 | | | | | | | | | 0/5 | | | | | 4/5 | | | | | **II** | **Y** |
| Westert et al.,^26^ | 1 | 2/2 | | 5/9 | | | | | | | | | 1/5 | | | | | 4/5 | | | | | **II** | **Y** |
| Roberts et al.,^25^ | 1 | 2/2 | | 6/9 | | | | | | | | | 5/5 | | | | | 5/5 | | | | | **I** | **N** |
| Momiyama et al.,^19^ | 1 | 2/2 | | 6/9 | | | | | | | | | 4/5 | | | | | 5/5 | | | | | **I** | **N** |
| Ellis et al.,^23^ | 1 | 2/2 | | 6/9 | | | | | | | | | 4/5 | | | | | 4/5 | | | | | **II** | **N** |
| Varela and Nguyen,^42^ | 1 | 2/2 | | 6/9 | | | | | | | | | 2/5 | | | | | 3/5 | | | | | **II** | **Y** |
| Poulose et al.,^39^ | 1 | 2/2 | | 5/9 | | | | | | | | | 3/5 | | | | | 3/5 | | | | | **II** | **Y** |
| Greenstein et al.,^27^ | 1 | 2/2 | | 8/9 | | | | | | | | | 5/5 | | | | | 5/5 | | | | | **I** | **Y** |
| Neureuther et al.,^48^ | 1 | 2/2 | | 6/9 | | | | | | | | | 4/5 | | | | | 3/5 | | | | | **II** | **Y** |
| Petrelli et al.,^28^ | 1 | 2/2 | | 6/9 | | | | | | | | | 5/5 | | | | | 4/5 | | | | | **I** | **Y** |
| McNabb-Baltar et al.,^53^ | 1 | 2/2 | | 6/9 | | | | | | | | | 5/5 | | | | | 4/5 | | | | | **I** | **Y** |
| Chang et al.,^21^ | 1 | 2/2 | | 4/9 | | | | | | | | | 2/5 | | | | | 2/5 | | | | | **III** | **N** |
| Roberts et al.,^24^ | 1 | 2/2 | | 6/9 | | | | | | | | | 4/5 | | | | | 5/5 | | | | | **I** | **Y** |
| Hanmer et al.,^38^ | 1 | 2/2 | | 6/9 | | | | | | | | | 5/5 | | | | | 4/5 | | | | | **I** | **Y** |
| Roberts et al.,^55^ | 1 | 2/2 | | 7/9 | | | | | | | | | 5/5 | | | | | 5/5 | | | | | **I** | **Y** |
| Loehrer et al.,^29^ | 1 | 2/2 | | 7/9 | | | | | | | | | 5/5 | | | | | 5/5 | | | | | **I** | **Y** |
| Palsson and Sandblom,^41^ | 1 | 2/2 | | 4/9 | | | | | | | | | 3/5 | | | | | 3/5 | | | | | **II** | **Y** |
| Compagnucci et al.,^22^ | 1 | 2/2 | | 6/9 | | | | | | | | | 4/5 | | | | | 4/5 | | | | | **II** | **N** |
| Mador et al., ^36^ | 1 | 2/2 | | 8/9 | | | | | | | | | 5/5 | | | | | 5/5 | | | | | **I** | **Y** |
| Ibrahim et al.,^50^ | 1 | 2/2 | | 7/9 | | | | | | | | | 5/5 | | | | | 5/5 | | | | | **I** | **Y** |
| Ambur et al.,^43^ | 1 | 2/2 | | 6/9 | | | | | | | | | 5/5 | | | | | 5/5 | | | | | **I** | **Y** |
| Lu et al.,^52^ | 1 | 2/2 | | 6/9 | | | | | | | | | 5/5 | | | | | 5/5 | | | | | **I** | **Y** |
| Kang et al.,^20^ | 1 | 2/2 | | 6/9 | | | | | | | | | 4/5 | | | | | 5/5 | | | | | **I** | **Y** |
| Lu et al.,^51^ | 1 | 2/2 | | 7/9 | | | | | | | | | 5/5 | | | | | 5/5 | | | | | **I** | **Y** |
| Bhutiani et al.,^31^ | 1 | 2/2 | | 6/9 | | | | | | | | | 5/5 | | | | | 4/5 | | | | | **I** | **Y** |
| Carmichael et al.,^45^ | 1 | 2/2 | | 6/9 | | | | | | | | | 4/5 | | | | | 4/5 | | | | | **II** | **Y** |
| Huang et al.^54^ | 1 | 2/2 | | 7/9 | | | | | | | | | 5/5 | | | | | 5/5 | | | | | **I** | **Y** |
| Moore et al.,^47^ | 1 | 2/2 | | 6/9 | | | | | | | | | 4/5 | | | | | 4/5 | | | | | **II** | **Y** |
| Carmichael et al.,^46^ | 1 | 1/2 | | 6/9 | | | | | | | | | 4/5 | | | | | 3/5 | | | | | **II** | **Y** |
| Schneider et al.,^44^ | 1 | 2/2 | | 6/9 | | | | | | | | | 5/5 | | | | | 5/5 | | | | | **I** | **Y** |
| Godat et al.,^30^ | 1 | 2/2 | | 7/9 | | | | | | | | | 5/5 | | | | | 4/5 | | | | | **I** | **Y** |
| McCarty et al.,^37^ | 1 | 2/2 | | 6/9 | | | | | | | | | 4/5 | | | | | 5/5 | | | | | **I** | **Y** |
| Janeway et al.,^49^ | 1 | 2/2 | | 6/9 | | | | | | | | | 4/5 | | | | | 5/5 | | | | | **I** | **Y** |
| Chouairi et al.,^35^ | 1 | 2/2 | | 6/9 | | | | | | | | | 4/5 | | | | | 5/5 | | | | | **I** | **Y** |
| Kabaria et al.,^40^ | 1 | 2/2 | | 7/9 | | | | | | | | | 4/5 | | | | | 4/5 | | | | | **I** | **Y** |
| Shmelev et al.,^34^ | 1 | 2/2 | | 6/9 | | | | | | | | | 4/5 | | | | | 5/5 | | | | | **I** | **Y** |

The items on the STROBE checklist v4 have here been interpreted in terms of their appropriateness of design to answer the study question. Scores were summarized as 0-11 = III, 12-17 = II, 18-22 = I, with one representing the highest quality studies.

Y: yes; N: No
