# Supplementary material for: Systematic Review: Impact of Social Determinants of Health on the Management and Prognosis of Gallstone Disease
Source: Health Equity. 2022 Oct 27;6(1):819–35. doi: 10.1089/heq.2022.0063 (PMC9629913; doi:10.1089/heq.2022.0063)
Supplement: Supplemental data [file Suppl_Data.zip › DQWRLF7P_64F1-DBBE-9482-132B-F637.pdf]

Editing Certificate

This document certifies that the manuscript

IMPACT OF SOCIO-ECONOMIC AND TERRITORIAL DETERMINANTS ON MANAGEMENT AND PROGNOSIS OF GALLSTONE DISEASE: A SYSTEMATIC REVIEW

prepared by the authors

Benoît Dupont1,2, Olivier Dejardin2,3, Véronique Bouvier2,3, Marie-Astrid Piquet1, Arnaud Alves2,4

was edited for proper English language, grammar, punctuation, spelling, and overall style by one or more of the highly qualified native English speaking editors at AJE.

This certificate was issued on **December 30, 2021** and may be verified on the AJE website using the verification code **64F1-DBBE-9482-132B-F637** .

Neither the research content nor the authors' intentions were altered in any way during the editing process. Documents receiving this certification should be English-ready for publication; however, the author has the ability to accept or reject our suggestions and changes. To verify the final AJE edited version, please visit our verification page at aje.com/certificate. If you have any questions or concerns about this edited document, please contact AJE at support@aje.com.

AJE provides a range of editing, translation, and manuscript services for researchers and publishers around the world. For more information about our company, services, and partner discounts, please visit aje.com.
